# Trends in incidence, mortality, and conditional survival of anaplastic thyroid cancer over the last two decades in the USA

**DOI:** 10.3389/fendo.2025.1585679

**Published:** 2025-06-04

**Authors:** Hongpeng Guo, Junjie Zhang, Yuanji Jia, Zongfeng Liu, Ying Qi, Chenglin Sun, Zhencun Cai, Ji Wu

**Affiliations:** ^1^ Department of General Surgery, Central Hospital Affiliated to Shenyang Medical College, Shenyang, Liaoning, China; ^2^ Department of Pathology, Central Hospital Affiliated to Shenyang Medical College, Shenyang, Liaoning, China; ^3^ Department of Orthopedics Surgery, The Second Hospital Affiliated to Shenyang Medical College, Shenyang, Liaoning, China; ^4^ School of Public Health, Shenyang Medical College, Shenyang, Liaoning, China

**Keywords:** anaplastic thyroid carcinoma, Epidemiology, SEER database, conditional survival, nomogram

## Abstract

**Background:**

Anaplastic thyroid carcinoma (ATC) is a highly aggressive malignancy, and there is currently a lack of up-to-date epidemiological data. Traditional survival analysis fails to capture the dynamic changes in prognosis for long-term survivors, while conditional survival (CS) analysis, a critical tool for adaptive risk stratification, remains underexplored in ATC.

**Methods:**

Patients diagnosed with ATC between 2000 and 2021 were identified from the Surveillance, Epidemiology, and End Results (SEER) database. Temporal trends in age-adjusted incidence and incidence-based mortality were analyzed using Joinpoint regression to calculate annual percentage changes (APCs) with 95% confidence intervals (CIs). Overall survival (OS) was estimated using the Kaplan-Meier method. CS rates were calculated using the formula: CS(y/x) = OS(y+x)/OS(x). Prognostic factors were identified using Best Subset Regression (BSR), LASSO, and univariate and multivariate Cox regression analyses, and these factors were incorporated into a CS-nomogram model. The predictive performance of the model was validated using evaluation metrics, including the area under the receiver operating characteristic curve (AUC). Point values were assigned to the model’s predictive factors, and a risk stratification system was developed based on the optimal threshold of the total score.

**Results:**

From 2000 to 2021, the age-adjusted incidence of ATC increased from 0.066 to 0.077 per 100,000 (APC: 2.308%, 95% CI: 1.187–3.441), peaking at 0.119 in 2018. Mortality trends paralleled this rise, with age-adjusted mortality increasing from 0.037 to 0.051 per 100,000 (APC: 2.380%, 95% CI: 1.129–3.646). CS analysis demonstrated a progressive increase in survival rates over time, with the 24-month cumulative survival rate rising from 14.0% to 93.8%, with the most pronounced temporal changes observed in patients with distant disease. Prognostic factors identified through BSR, LASSO, and Cox regression included age, SEER stage, and treatment. A novel CS-nomogram was successfully developed and validated for dynamic real-time survival prediction, enabling identification of high- and low-risk patient groups.

**Conclusion:**

The incidence and incidence-based mortality of ATC have increased over the past few decades. The CS rates of ATC patients have dynamically improved over time. The CS-nomogram, integrating age, SEER stage, and treatment, provides clinicians with a personalized, dynamic, and real-time survival prediction tool that helps alleviate survivors’ psychological distress, reduces anxiety, and optimizes precision follow-up strategies.

## Introduction

The global incidence of thyroid cancer has increased threefold over the past four decades (from 5.0 to 14.6 per 100,000), while mortality rates have paradoxically remained stagnant at 0.5 per 100,000, sparking debates about overdiagnosis (1). Anaplastic thyroid cancer (ATC) is a rare but highly aggressive malignancy, accounting for only 1–2% of thyroid cancers, yet responsible for more than 50% of thyroid cancer-related deaths, with a median overall survival (OS) of only 3 to 5 months (2–4). Unfortunately, the current epidemiological data on ATC remain outdated; available statistics extend only through 2015 or earlier (5–8) and therefore lack the most recent information on this aggressive cancer.

Due to the rarity and aggressive nature of ATC, patients often experience heightened anxiety regarding their prognosis following diagnosis. Conventional survival models, such as the AJCC TNM staging system, typically offer static survival predictions based solely on initial staging parameters (9). Although contemporary nomograms have integrated additional clinicopathological variables for predicting ATC survival, these models remain fundamentally limited in their ability to capture the temporal evolution of survival probabilities (10–12). This limitation proves particularly consequential in aggressive malignancies like ATC, where approximately 80% of mortality events cluster within the first year post-diagnosis. To address this critical gap, conditional survival (CS) methodology emerges as an innovative prognostic tool that dynamically recalibrates survival estimates based on accrued survival time (13–15). By integrating both baseline prognostic factors and the duration of survival achieved, CS not only provides a time-adaptive risk assessment that may alleviate psychological distress in survivors who have overcome the initial high-risk period, but also enables dynamic risk stratification to inform personalized surveillance protocols and therapeutic decision-making.

In this study, we aim to investigate the latest epidemiological evidence of ATC using the Surveillance, Epidemiology, and End Results (SEER) database from the National Cancer Institute (NCI) and explore the dynamic changes in survival probabilities over time through CS analysis. Furthermore, we developed and validated the first CS-based prognostic nomogram for ATC patients, which provides real-time updated survival estimates, enhances personalized risk stratification, and optimizes follow-up scheduling for better patient management.

## Methods

### Data source

This study utilized the SEER Incidence Research Data, 17 Registries, Nov 2023 Subset (2000–2021) for incidence cases and CS analyses, while mortality data for incidence-based mortality (IBM) analysis were obtained from the SEER IBM Research Data, 17 Registries, Nov 2023 Subset (2000–2021). SEER database, a comprehensive resource managed by the National Cancer Institute, covers approximately 34.6% of the U.S. population through 17 population-based cancer registries, providing valuable incidence and survival data for epidemiological analyses (16, 17), which were accessed using SEER*Stat software (version 8.4.4; seer.cancer.gov/seerstat). Since the SEER database is publicly accessible and contains de-identified data, this study was exempt from ethical approval by the Ethics Committee of Central Hospital Affiliated to Shenyang Medical College, and adhered to the Strengthening the Reporting of Observational Studies in Epidemiology (STROBE) guidelines (18).

### Study population

Patients diagnosed with ATC were identified using the ICD-O-3 codes (C73.9) for site and the ICD-O-3 histology codes (8021) for histological classification. For incidence analysis, cases diagnosed between 2000 and 2021 with diagnostic confirmation via positive histology were included; IBM analysis further restricted to cases with ‘One primary only’ in sequence number and excluded those reported solely by death certificates or autopsy. CS analysis excluded patients with unknown survival data, 0-month survival time, missing SEER combined summary stage, or incomplete treatment records (surgery, radiation, chemotherapy).

### Clinical variables

The clinical variables analyzed in this study, obtained from the SEER database, included patient demographics (age, sex, race), SEER combined summary stage (localized, regional, distant), and treatment history (surgery, radiation, chemotherapy). Disease staging followed the SEER combined summary criteria, categorizing tumors as localized (restricted to the thyroid), regional (propagated beyond the thyroid to the surrounding tissue or lymph nodes), or distant (metastasized to other organs) (19). Radiotherapy was defined per SEER criteria as receipt of ‘beam radiation’ (radiation targeted to malignant tissue). Treatment strategies were classified into eight categories based on the receipt of surgery, radiation therapy (RT), and/or chemotherapy (CT): Untreated, Only RT, Only CT, Only Surgery, Surgery+CT, Surgery+RT, RT+CT, and Surgery+RT+CT.

### Overall and conditional survival analysis

The primary endpoint was OS, defined as the time from diagnosis to death from any cause, with living patients censored at last follow-up. OS was estimated via the Kaplan-Meier method, with comparisons between subgroups assessed by log-rank tests. CS, a dynamic prognostic metric reflecting the evolving likelihood of survival after surpassing initial time thresholds, was calculated as: CS(y/x) = OS(y+x)/OS(x) (20), where OS(x) and OS(x+y) denote the Kaplan-Meier survival probabilities at x years and x+y years post-diagnosis, respectively.

### CS-nomogram construction and validation

The full cohort of ATC patients was randomly divided into training and validation groups in a 7:3 ratio for the construction and validation of the CS-Nomogram. In the training group, prognostic variables for ATC patients were comprehensively selected using three methods: Best Subset Regression (BSR), Least Absolute Shrinkage and Selection Operator (LASSO), and univariate and multivariate Cox regression. The BSR method systematically evaluated all possible combinations of variables by exhaustively testing each combination, with the optimal variables selected based on the highest adjusted R^2^ value. LASSO, combined with strict 10-fold cross-validation, determined key variables based on the lambda-1se value. Variables with a P-value < 0.05 in the univariate Cox model were further analyzed in the multivariate Cox regression. Variables consistently identified by all three methods were integrated into the CS-nomogram.

### Statistical analysis

Continuous variables following a normal distribution are presented as the mean ± standard deviation (SD). Continuous variables with skewed distributions are expressed as medians with interquartile ranges (IQR). Categorical variables are presented as frequencies and corresponding percentages. The age-adjusted incidence and IBM rates were calculated using SEER*Stat software, version 8.4.4. The annual percentage change (APC) was determined by fitting a linear regression model. This involved regressing the logarithm of the age-adjusted rates over time, with the resulting slope transformed to calculate the yearly percentage change. Survival analyses were conducted using R software (version 4.1.3) and the Free Statistics software (version 2.1). A P-value < 0.05 was considered indicative of statistical significance in all analyses.

## Results

### Annual incidence and mortality trends of ATC from 2000 to 2021

**Figure 1:**
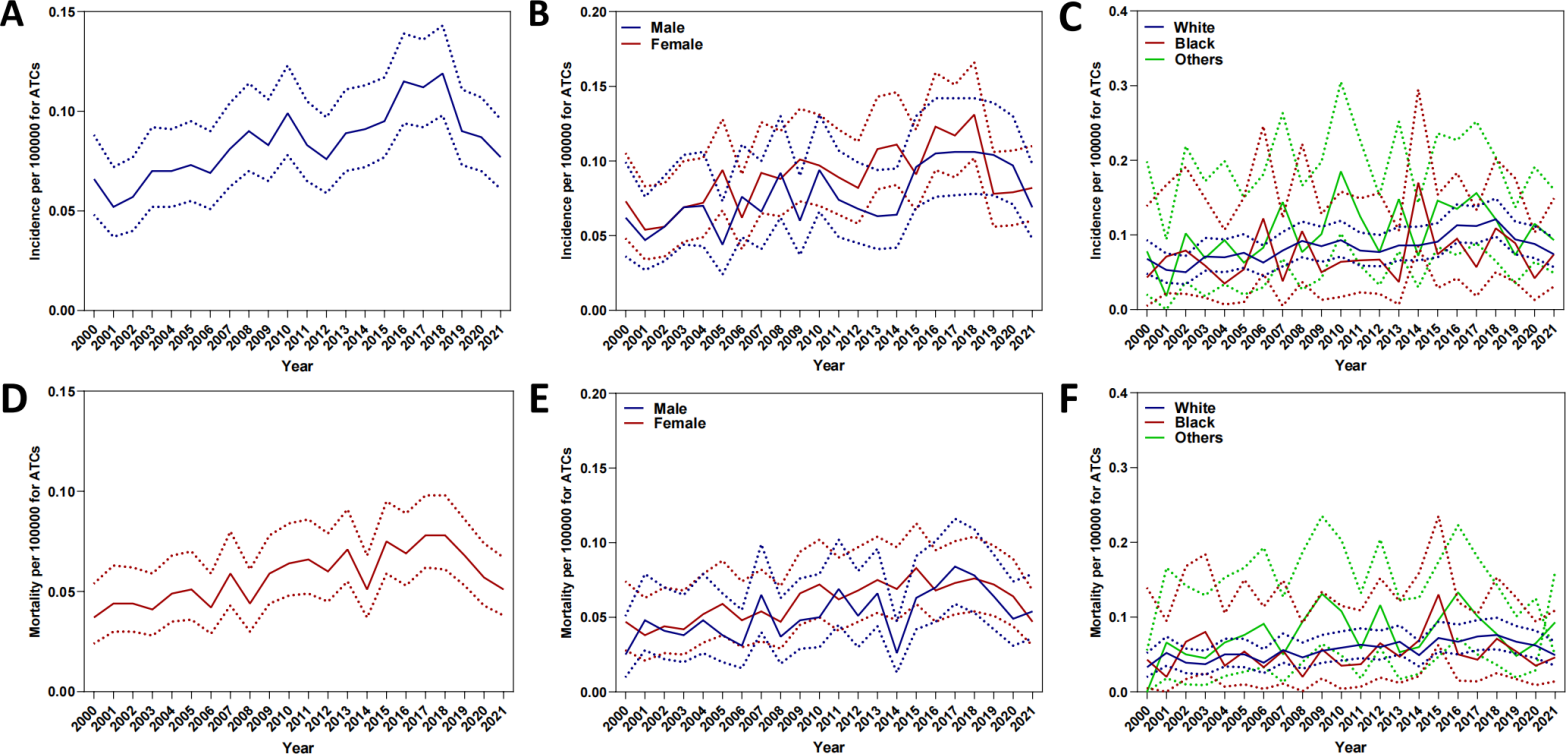
Annual age-adjusted incidence and mortality of ATC (2000–2021). **(A)** Overall incidence; **(B)** Incidence by sex. **(C)** Incidence by race. **(D)** Overall mortality. **(E)** Mortality by sex. **(F)** Mortality by race.

From 2000 to 2021, the age-adjusted incidence of ATC exhibited a significant upward trajectory, rising from 0.066 to 0.077 per 100,000 (APC = 2.308%, 95% CI: 1.187–3.441), with a pronounced peak in 2018 (0.119 per 100,000) (**Figure 1A**). Sex-stratified analyses revealed persistently higher acceleration in males (APC = 2.718%, 95% CI: 1.296–4.161) compared to females (APC = 2.081%, 95% CI: 0.598–3.585) (**Figure 1B**). Racial disparities were pronounced, with White individuals driving the majority of the increase (APC = 2.390%, 95% CI: 1.223–3.569), while Black (APC = 1.369%, 95% CI: –1.713–4.547) and Others (APC = 1.303%, 95% CI: –1.334–4.011) racial groups exhibited non-significant trends (**Figure 1C**).

Mortality trends paralleled rising incidence, with age-adjusted rates increasing from 0.037 to 0.051 per 100,000 (APC = 2.380%, 95% CI: 1.129–3.646) (**Figure 1D**). Sex-stratified analyses revealed accelerated mortality escalation in males (APC = 2.773%, 95% CI: 0.852–4.732) compared to females (APC = 2.154%, 95% CI: 0.886–3.438) (**Figure 1E**). With regard to racial disparities (**Figure 1F**), the mortality rate for White individuals increased from 0.033 to 0.049 per 100,000, corresponding to an APC of 2.477% (95% CI: 1.287–3.680%). Meanwhile, the APC for Black individuals was 0.779% (95% CI: –2.374–4.034%), and for Others, the APC was 0.078% (95% CI: –0.019–0.019%).

### Baseline characteristics

Baseline characteristics of the study cohort are summarized in **Table 1**. Between 2000 and 2021, a total of 946 ATC patients were included and randomly divided into a training set (n=662) and a validation set (n=284). The mean age at diagnosis was 67.5 years, with a higher proportion of females (56.7%) than males (43.3%), and 79.5% of the patients were White. At diagnosis, 67.2% presented with distant metastasis. In terms of treatment, with 26.7% of patients receiving combination therapy (Surgery + RT + CT), while 10.7% did not receive any treatment.

**Table 1:** Patients demographic and clinical characteristics.

Characteristic	Overall (N=946)	Training (N=662)	Validation (N=284)	*P*
Age, Mean ± SD	67.5 (12.0)	67.6 (12.2)	67.1 (11.5)	0.583
Sex, n (%)				0.247
Male	410 (43.3%)	295 (44.6%)	115 (40.5%)	
Female	536 (56.7%)	367 (55.4%)	169 (59.5%)	
Race, n (%)				0.761
White	752 (79.5%)	523 (79.0%)	229 (80.6%)	
Black	69 (7.3%)	48 (7.3%)	21 (7.4%)	
Other/unknown	125 (13.2%)	91 (13.7%)	34 (12.0%)	
SEER Stage, n (%)				0.719
Localized	67 (7.1%)	46 (6.9%)	21 (7.4%)	
Regional	243 (25.7%)	175 (26.4%)	68 (23.9%)	
Distant	636 (67.2%)	441 (66.6%)	195 (68.7%)	
Treatment, n (%)				0.646
Untreated	101 (10.7%)	69 (10.4%)	32 (11.3%)	
Only RT	102 (10.8%)	67 (10.1%)	35 (12.3%)	
Only CT	34 (3.6%)	21 (3.2%)	13 (4.6%)	
Only Surgery	136 (14.4%)	94 (14.2%)	42 (14.8%)	
Surgery+CT	47 (5.0%)	35 (5.3%)	12 (4.2%)	
Surgery+RT	89 (9.4%)	59 (8.9%)	30 (10.6%)	
RT+CT	184 (19.5%)	137 (20.7%)	47 (16.5%)	
Surgery+RT+CT	253 (26.7%)	180 (27.2%)	73 (25.7%)	

RT, Radiation; CT, Chemotherapy.

### Overall and conditional survival

**Figure 2:**
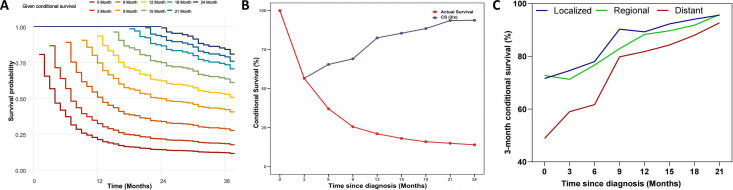
Conditional-survival dynamics in ATC. **(A)** Kaplan-Meier curves showing conditional survival (y-axis) for patients who had already survived 0, 3, 6, 9, 12, 15, 18, 21, or 24 months after diagnosis (x-axis). **(B)** CS(3/x) curve showing the probability of survival another 3 months after surviving for x months. **(C)** 3-month conditional survival stratified by SEER stage: localized, regional, and distant.

The median follow-up duration was 4 months (interquartile range, 2-9 months). Among the 946 patients, 826 (87.3%) died during the follow-up period. The 6-, 12-, and 24-month OS rates of the overall population were 37.1%, 21.1% and 14.0%, respectively (**Supplementary Figure S1A**). The 6-month and 12-month OS rates for patients with localized, regional, and distant disease were as follows: 58% and 43% for localized disease, 51.9% and 33% for regional disease, and 28% and 14% for distant disease, respectively(**Supplementary Figure S1B**). CS analysis demonstrated a progressive increase in cumulative survival over time (**Figure 2A**). The 24-month cumulative survival rate increased incrementally from 14.0% to 24.8%, 37.9%, 54.8%, 66.5%, 77.8%, 87.8%, and ultimately reached 93.8% after surviving 3 to 21 months (**Table 2**). The CS(3|x) curve shows that as patients accumulate survival time, the probability of surviving an additional 3 months progressively increases (**Figure 2B**). In addition, the CS showed more significant changes over time in patients with distant disease. The 3-month CS at different time points for patients with various SEER stages is shown in **Figure 2C**.

**Table 2:** The conditional survival for ATC patients at a given time point.

Already survival months	Total survival time							
	3 months	6 months	9 months	12 months	15 months	18 months	21 months	24 months
0 month	56.6%	37.1%	25.6%	21.1%	18.1%	16.0%	15.0%	14.0%
3 months		65.5%	45.2%	37.3%	31.9%	28.2%	26.4%	24.8%
6 months			69.1%	57.0%	48.7%	43.1%	40.4%	37.9%
9 months				82.5%	70.5%	62.4%	58.5%	54.8%
12 months					85.5%	75.7%	70.9%	66.5%
15 months						88.5%	82.9%	77.8%
18 months							93.6%	87.8%
21 months								93.8%

### Construction a prognostic CS nomogram

**Figure 3:**
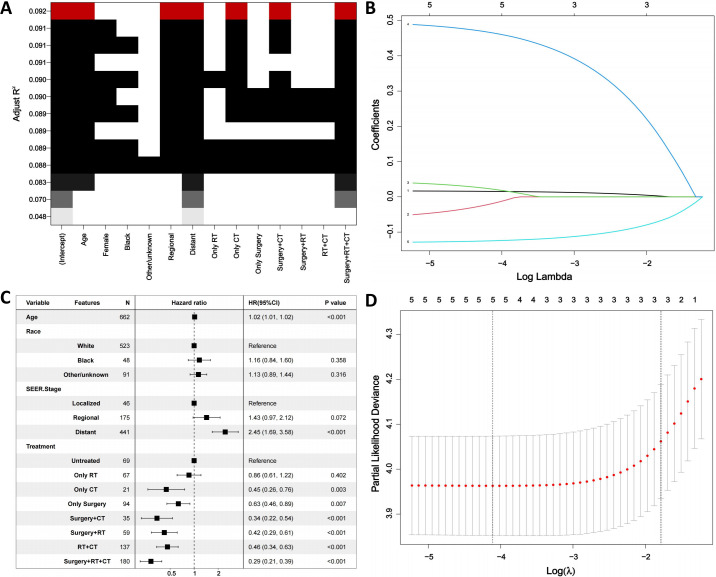
Identification of prognostic factors. **(A)**. Best subset regression (BSR). **(B, D)**. The least absolute shrinkage and selection operator (LASSO) regression. **(C)**. Multivariable analysis. RT, Radiation; CT, Chemotherapy.

**Figure 4:**
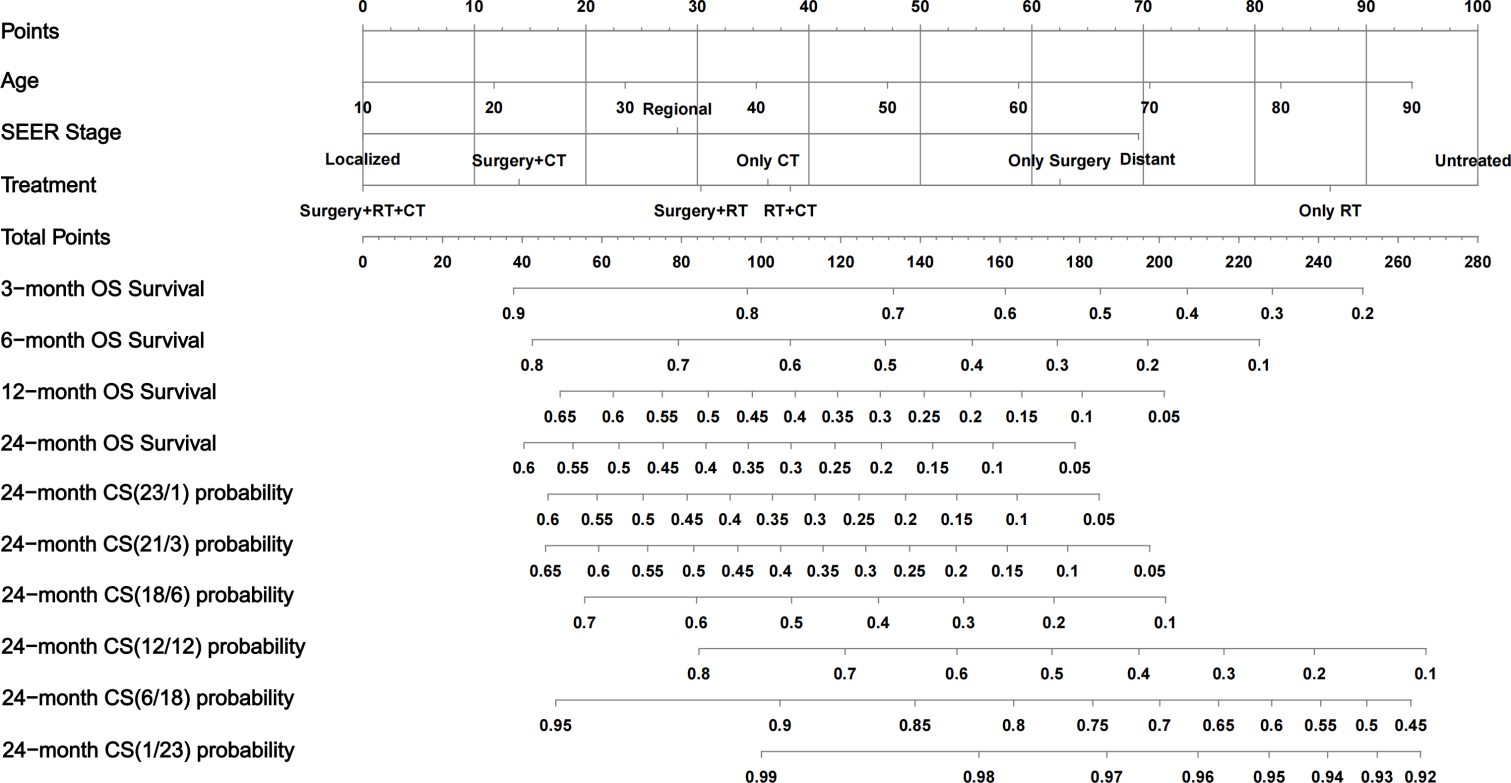
Conditional-survival nomogram for ATC patients. The nomogram incorporates three prognostic factors—Age, SEER stage, and Treatment. Draw a vertical line from each patient’s value to the Points scale. Sum the three point values to obtain the Total Points. Project downward to read predicted overall-survival probabilities at 3, 6, 12, and 24 months and multiple 24-month CS probabilities. OS, overall survival; CS, conditional survival; RT, radiotherapy; CT, chemotherapy.

First, the BSR model was used to identify variables influencing OS in ATC patients, with age, SEER stage, and treatment emerging as key prognostic factors (**Figure 3A**). Next, the LASSO regression method was employed to further refine the selection of variables, and age, SEER stage, and treatment were consistently identified as major prognostic factors (**Figures 3B, D**). Subsequently, univariate Cox regression analysis was conducted to identify variables associated with OS, including age, race, SEER stage, and treatment (**Table 3**). These variables were then included in a multivariate Cox regression model, confirming that age, SEER stage, and treatment modality were independent prognostic factors (**Table 3**; **Figure 3C**). Following this, we incorporated CS into the nomogram model, using these selected variables to develop a CS-based prediction tool (**Figure 4**). Compared to traditional survival prediction models, the CS-nomogram not only predicted 3-, 6-, 12-, and 24-month OS based on personalized clinicopathological features but also provided 24-month CS predictions, contingent upon the number of months the patients had survived post-diagnosis.

**Table 3:** Univariate and multivariate cox regression analysis of factors influencing overall survival.

Characteristic	Univariate analysis		Multivariable analysis	
	HR (95%CI)	*P*	HR (95%CI)	*P*
Age	1.02 (1.01-1.03)	<0.001	1.02 (1.01-1.02)	<0.001
Sex				
Male				
Female	1.05 (0.89-1.23)	0.597		
Race				
White				
Black	1.10 (0.80-1.51)	0.547	1.16 (0.84-1.60)	0.358
Other/unknown	1.28 (1.01-1.63)	0.042	1.13 (0.89-1.44)	0.316
SEER Stage				
Localized				
Regional	1.40 (0.95-2.07)	0.085	1.43 (0.97-2.12)	0.072
Distant	2.43 (1.69-3.51)	<0.001	2.45 (1.69-3.58)	<0.001
Treatment				
Untreated				
Only RT	0.93 (0.66-1.31)	0.695	0.86 (0.61-1.22)	0.402
Only CT	0.39 (0.23-0.67)	<0.001	0.45 (0.26-0.76)	0.003
Only Surgery	0.50 (0.36-0.69)	<0.001	0.63 (0.46-0.89)	0.007
Surgery+CT	0.32 (0.20-0.50)	<0.001	0.34 (0.22-0.54)	<0.001
Surgery+RT	0.34 (0.24-0.50)	<0.001	0.42 (0.29-0.61)	<0.001
RT+CT	0.43 (0.32-0.58)	<0.001	0.46 (0.34-0.63)	<0.001
Surgery+RT+CT	0.23 (0.17-0.31)	<0.001	0.29 (0.21-0.39)	<0.001

OS, Overall Survival; RT, Radiation; CT, Chemotherapy.

### CS-nomogram validation

**Figure 5:**
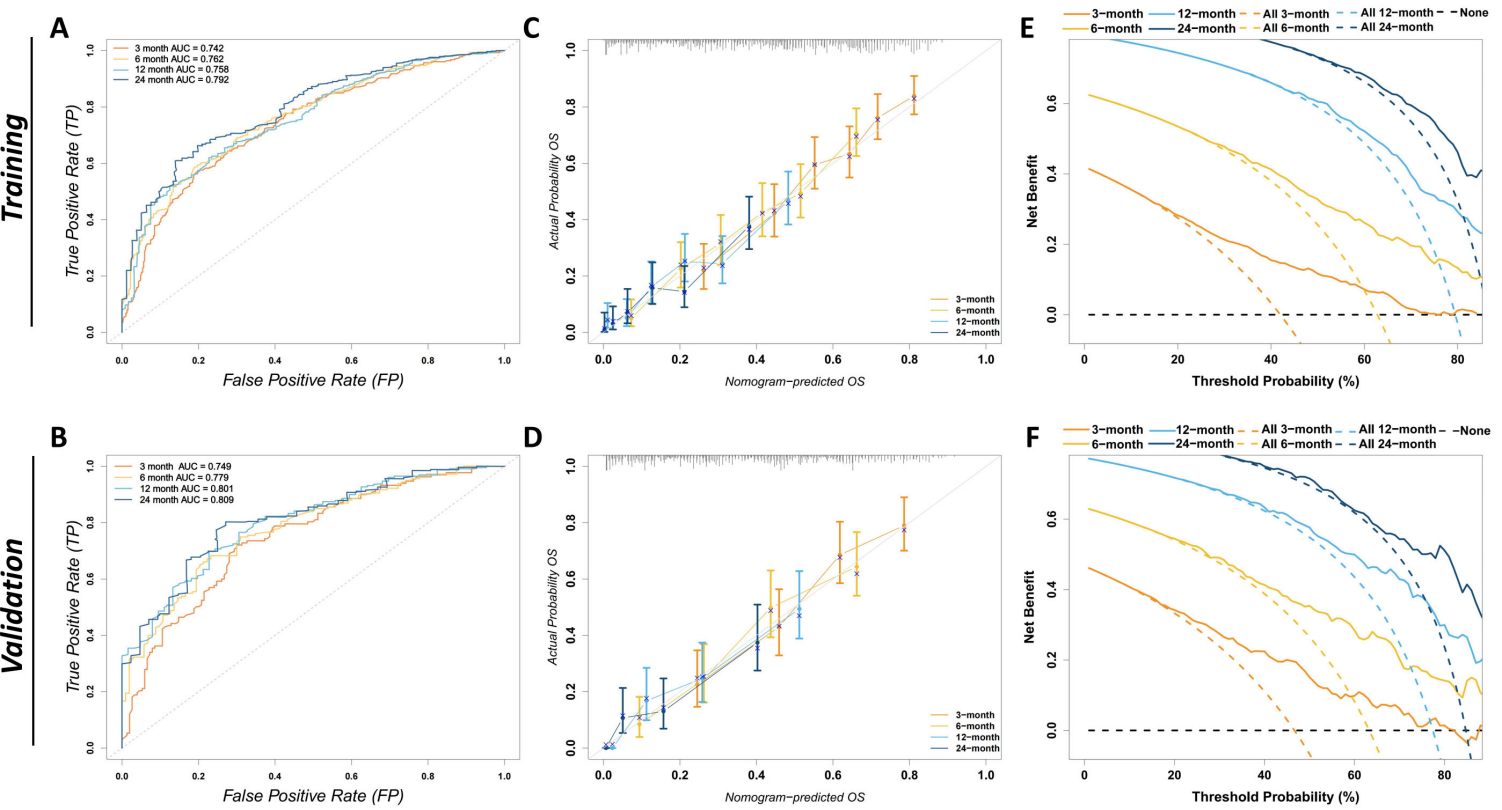
Evaluation of the discriminatory power, calibration, and clinical utility of the CS-nomogram. **(A)** The ROC curves for the training cohort depict the model’s ability to predict overall survival at 3, 6, 12, and 24 months, with corresponding AUCs of 0.742, 0.762, 0.758, and 0.792. An AUC closer to 1 indicates greater discriminative power. The x-axis represents the false-positive rate and the y-axis the true-positive rate. **(B)** The ROC curves for the validation cohort show AUCs of 0.749, 0.779, 0.801, and 0.809 for 3, 6, 12, and 24 months, respectively, confirming the model’s consistent discriminative performance in an independent dataset. **(C)** Calibration plots in the training cohort for 3-, 6-, 12-, and 24-month overall survival. The x-axis shows nomogram-predicted probability, and the y-axis the observed survival. The grey 45° reference line denotes perfect calibration; error bars indicate bootstrap-derived 95% confidence intervals. **(D)** Calibration plots for the validation cohort, confirming good agreement between predicted and observed survival probabilities. **(E)** DCA in the training cohort. The y-axis displays net benefit and the x-axis the threshold probability. Solid curves represent the CS nomogram at 3, 6, 12, and 24 months; color-matched dashed curves indicate a “treat-all” strategy, and the black dashed line a “treat-none” strategy. A nomogram curve that lies above both reference strategies indicates greater clinical benefit across that threshold range. **(F)** DCA for the validation cohort, corroborating the nomogram’s clinical utility in an external dataset. ROC, receiver-operating characteristic; AUC, area under the ROC curve; CS, conditional survival; DCA, decision-curve analysis; FP, false-positive rate; TP, true-positive rate; OS, overall survival.

The prognostic CS-nomogram model for predicting ATC patients was rigorously validated and evaluated in both the training and validation cohorts. In the training cohort, the model achieved a C-index of 0.695 (95% CI: 0.671–0.719), and in the validation cohort, it reached 0.708 (95% CI: 0.671–0.744). The model’s predictive performance was further assessed using the area under the receiver operating characteristic (AUC) curve. In the training cohort (**Figure 5A**), the AUCs at 3, 6, 12, and 24 months were 0.742, 0.762, 0.758, and 0.792, respectively. In the validation cohort (**Figure 5B**), the AUCs were 0.749, 0.779, 0.801, and 0.809. Calibration curves for the 3-, 6-, 12-, and 24-month OS nomograms in both the training (**Figure 5C**) and validation (**Figure 5D**) cohorts demonstrated excellent consistency between the predicted and actual survival. Finally, clinical decision curve analysis (DCA) revealed significant net benefits at 3, 6, 12, and 24 months, further highlighting the model’s clinical applicability and potential value (**Figures 5E, F**).

### CS-nomogram-based risk stratification

**Figure 6:**
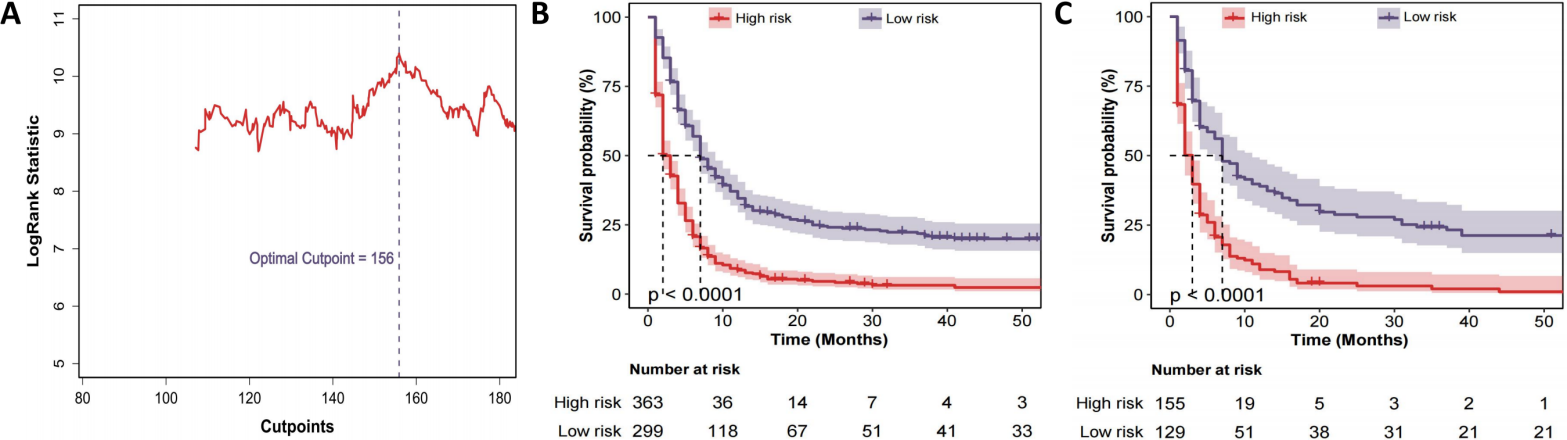
Risk-stratification derived from the CS-nomogram. **(A)** Log-rank statistic plotted against all possible total-point cut-points; the maximum statistic identifies 156 as the optimal threshold that separates patients into high- and low-risk groups. **(B)** Kaplan–Meier curves for overall survival in the training cohort stratified by this threshold (high risk; low risk). Numbers at risk are shown below the x-axis. **(C)** Kaplan–Meier curves for the validation cohort, demonstrating that the same 156-point cut-off reproduces a highly significant separation of survival in an independent dataset.

The CS-nomogram was used to calculate prognostic scores for %all patients. Based on the optimal cut-off value of 156 identified from the training cohort, patients were divided into low-risk and high-risk groups (**Figure 6A**). Kaplan-Meier survival analysis was then conducted to compare survival outcomes between the two groups in both the training and validation cohorts. The results showed significant survival differences between the low-risk and high-risk groups (**Figures 6B, C**), further confirming the CS-nomogram’s utility in risk stratification and survival prediction.

## Discussion

In this SEER database-based study, we provide the latest epidemiological analysis of ATC. From 2000 to 2021, age-adjusted incidence (APC = 2.308%) and mortality (APC = 2.380%) exhibited near-synchronized growth, with accelerated progression observed in males (incidence APC = 2.718% vs. 2.081% in females; mortality APC = 2.773% vs. 2.154%). White individuals dominated the increase in both incidence (APC = 2.390%) and mortality (APC = 2.477%). This contrasts with earlier SEER-based studies documenting stable ATC incidence during 1973-2002 and 1986-2015 (5, 21), as well as with reports from Europe and other regions of the world showing similarly plateaued incidence (7, 8, 22), suggesting a potential recent acceleration in disease trends. These epidemiological trends reflect the disproportionate rise in disease burden within specific populations, emphasizing the urgent need for targeted intervention strategies.

We present a large cohort of ATC patients and provide a comprehensive survival analysis.The OS data reflect the disease’s lethality, with 24-month OS rates of 14.0% for the entire cohort and 14% at 12 months for distant disease. Notably, our CS analysis reveals dynamic prognostic improvement: 24-month CS increased from 14.0% at diagnosis to 93.8% in patients already surviving 21 months. This aligns with prior observations by Xiang et al. (23), who reported that 1-year conditional cancer-specific survival in ATC rose incrementally with each additional survival year. Similarly, Yu et al. (24) highlighted the significant impact of tumor differentiation on prognosis, with ATC patients showing the highest mortality risk yet the greatest improvements in CS. Additionally, we found that the most significant improvement in conditional survival (CS) occurred in patients with distant ATC disease. This pattern is similar to what has been observed in distant oncocytic cell carcinoma (OCC) of the thyroid (19). Static OS metrics inadequately represent the time-dependent evolution of prognosis in aggressive malignancies, overlooking temporal dependency. CS estimates empower clinicians to progressively refine survival projections, offering critical decision-support for metastatic ATC patients who surpass early treatment phases.

Through BSR, LASSO, and univariate/multivariate Cox proportional hazards modeling, we identified age, SEER stage, and treatment as independent prognostic factors for ATC. Age remained a significant factor, as observed in prior studies (11, 25, 26). We also found that SEER stage was an important prognostic factor, as the risk of mortality for patients with distant metastasis was 2.45 times that of patients with localized primary tumors, which is similar to findings in previous studies (26–30). Additionally, Our analysis highlights the critical role of multimodal therapy. Previous studies on ATC treatment often employed single-variable analyses, which are prone to multicollinearity interference. By using combined treatment strategies, we capture the synergistic effects of different therapies. Compared to untreated patients, all treatment modalities, including monotherapy, significantly reduced the mortality risk. Among all treatment combinations, the combination therapy showed a hierarchical survival advantage: Surgery + RT + CT provided the strongest protection (HR = 0.29, 95% CI:0.21–0.39), followed by Surgery + CT (HR = 0.34, 95% CI:0.22–0.54) and RT + CT (HR = 0.46, 95% CI:0.34–0.63). These findings align with recent SEER-based studies. Zhou et al. (31) demonstrated that radiotherapy combined with chemotherapy (RCT) improved survival regardless of metastasis status (adjusted HR = 0.69 for OS). The survival benefit of surgery in advanced ATC deserves emphasis. While guidelines recommend surgery for early-stage ATC (stage IVA and IVB) (32), Guo et al. (33) and Yin et al. (34) reported that surgery combined with adjuvant radiation and chemotherapy improves OS in stage IVC ATC patients. Notably, chemotherapy emerged as a cornerstone of treatment—both as monotherapy (HR = 0.45) and in combinations. This aligns with the mechanistic rationale that cytotoxic agents may mitigate systemic progression. More prospective trials are needed to validate these multimodal strategies, particularly in the era of targeted and immunotherapy.

The nomogram stands out for its evidence-based, individualized, and precise risk evaluation, making it a valuable tool for survival prediction in various cancers. Previous studies have established prognostic nomograms for ATC by identifying key survival determinants (10, 11, 26, 35). However, the biggest limitation of these models is their static nature, as they rely solely on initial patient characteristics for prediction and fail to reflect real-time changes in disease progression and risk. We developed the first CS nomogram for ATC patients. Compared with previous static models, this CS-based nomogram continuously updates survival probability estimates according to the time already survived, providing more dynamic and individualized prognostic predictions. This dynamic approach captures the evolving prognosis of ATC patients during follow-up more accurately. The CS-based nomogram model demonstrated excellent predictive performance in both the training and validation cohorts, providing continuously updated survival probability estimates for ATC patients. Additionally, a key feature of this model is its ability to stratify risk by assigning risk scores to ATC patients. For high-risk patients with a total score > 156, follow-up examinations can be scheduled at shorter intervals after diagnosis, with earlier multidisciplinary reassessment. Conversely, for low-risk patients, the surveillance interval may be safely extended, reducing both cost and anxiety. In addition, the nomogram can generate an updated survival probability at every outpatient visit, offering objective data for survivorship management and psychological counseling. When a patient’s conditional survival improves markedly, this information can be shared to alleviate fear of recurrence and guide targeted psychological support. Finally, the nomogram can be integrated into the electronic medical record system so that automatic alerts are triggered whenever a patient’s conditional risk exceeds a predefined threshold, prompting timely treatment-planning discussions or referral to supportive-care services.

We acknowledge several limitations in this study. First, the retrospective nature of this study inherently risks selection bias, particularly due to exclusion of cases with incomplete treatment documentation, which may underrepresent real-world heterogeneity. Secondly, although leveraging the SEER database enabled the inclusion of a large cohort, detailed treatment information—such as specific chemotherapy regimens, radiation dosimetry, surgical intent, molecular profiling, targeted therapies, and immunotherapy—is unavailable. This lack of granularity limits our ability to perform treatment-specific survival analyses and could substantially affect the clinical interpretation of our findings. Third, the lack of external validation is an important limitation that affects the generalizability of our findings. Given the differences in healthcare systems, clinical practice patterns, population genetics, and resource availability, the predictive performance and applicability of our CS-nomogram in different healthcare settings or diverse populations remain uncertain. Future studies must validate this nomogram externally across multiple regions and healthcare systems to confirm its broader utility and robustness. Finally, the SEER database has limitations in the range of variables it includes, omitting factors that could impact the survival of ATC patients, such as access to healthcare, imaging features, and molecular biomarkers. Future efforts should prioritize prospective and multicenter cohorts, coupled with API-enabled platforms for dynamic model refinement and clinician engagement.

## Conclusions

ATC has shown an increasing incidence (APC = 2.308%) and mortality (APC = 2.380%) over the past two decades. CS analysis revealed dynamic prognostic improvements, particularly in distant disease. The novel CS-nomogram we developed, incorporating age, SEER stage, and treatment, enables real-time adaptive risk stratification, helping clinicians adjust surveillance intensity and reduce patient anxiety through precise prognostication.

## Data Availability

The raw data supporting the conclusions of this article will be made available by the authors, without undue reservation.

## Supplementary material

Supplementary Figure 1 Overall survival of ATC patients. **(A)**. Kaplan–Meier curve for the entire cohort, showing a rapid decline in survival within the first 6 months after diagnosis. **(B)**. Kaplan–Meier curves stratified by SEER stage—Localized, Regional, and Distant.Tick marks denote censored observations.
