# Supplementary figures and images for: Trends in incidence, mortality, and conditional survival of anaplastic thyroid cancer over the last two decades in the USA
Source: Front Endocrinol (Lausanne). 2025 Jun 4;16:1585679. doi: 10.3389/fendo.2025.1585679 (PMC12173906; doi:10.3389/fendo.2025.1585679)

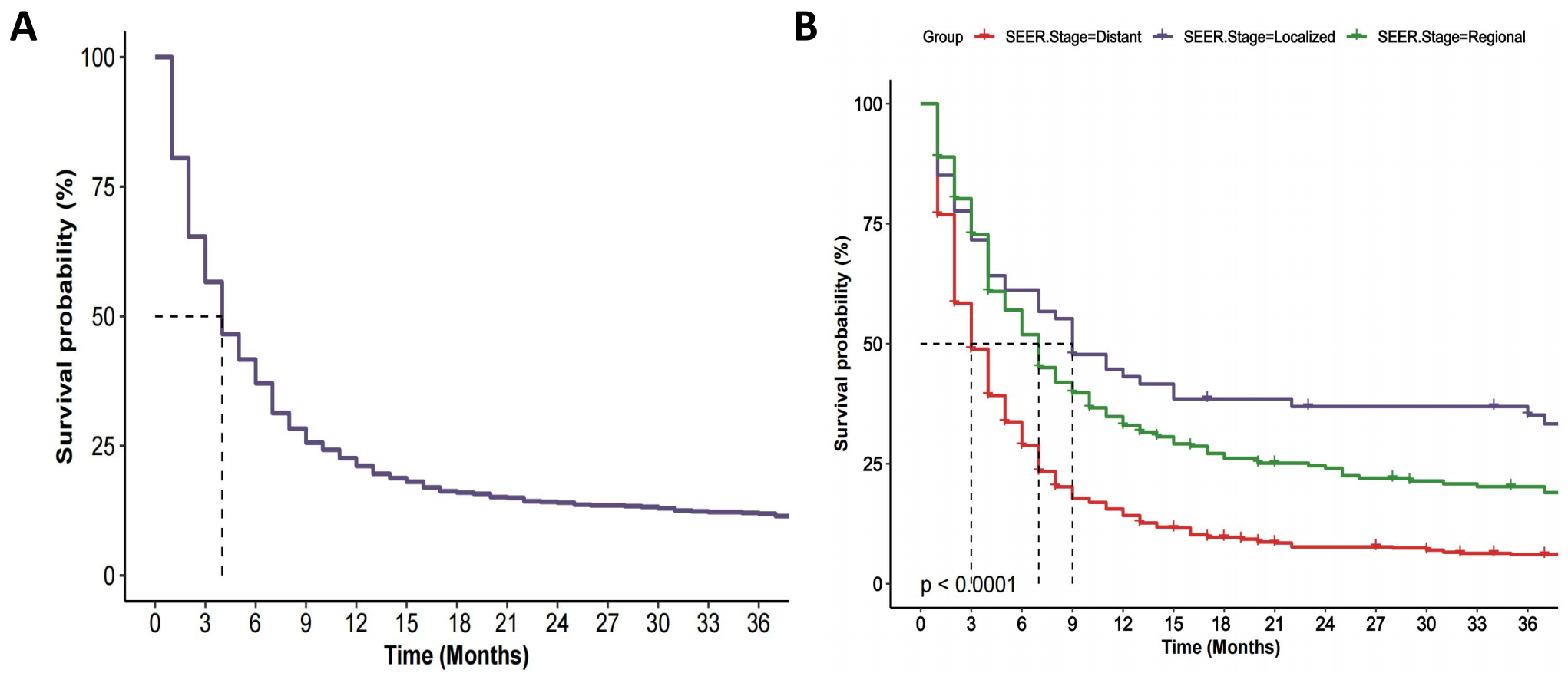

Supplement: Supplementary Figure 1 — Overall survival of ATC patients. (A). Kaplan–Meier curve for the entire cohort, showing a rapid decline in survival within the first 6 months after diagnosis. (B). Kaplan–Meier curves stratified by SEER stage—Localized, Regional, and Distant.Tick marks denote censored observations. [file Image1.tif]
